# Supplementary material for: Molecular Alterations in Sporadic and SOD1-ALS Immortalized Lymphocytes: Towards a Personalized Therapy
Source: Int J Mol Sci. 2021 Mar 16;22(6):3007. doi: 10.3390/ijms22063007 (PMC8000750; doi:10.3390/ijms22063007)
Supplement: Supplementary file 1 [file ijms-22-03007-s001.pdf]

Table S1. List of primers used in this study.

Gene product	Forward primer	Reverse primer
β-ACTIN	5′ TCCTTCCTGGGCATGGAG 3′	5′ AGGAGGAGCAATGATCTTGATCTT 3′
HMOX1	5′ TGCTCAACATCCAGCTCTTTGA 3′	5′ GCAGAATCTTGCACTTTGTTGCT 3′
IL-1β	5′ CTGGTGTGTGACGTTCCCATTA 3′	5′CCGACAGCACGAGGCTTT 3′
IL-6	5′CCTACCCCAATTTCCAATGCT3′	5′ TATTTTCTGACCACAGTGAGGAATG 3′
NFE2L2	5′CCCGAAGCACGCTGAAGGCA 3′	5′ CCAGGCGGTGGGTCTCCGTA 3′
NQO1	5′ GTTCATAGGAGAGTTTGCTT 3′	5′ TAGAACCTCAACTGACACTT 3′
TBP	5′ TGCACAGGAGCCAAGAGTGAA 3′	5′ CACATCACAGCTCCCCACCA 3′
TNF	5′ CATCTTCTCAAAATTCGAGTGACAA 3′	5′ TGGGAGTAGACAAGGTACAACCC 3′
TXN	5′ TTTCAGGAAGCCTTGGACGCT 3′	5′ GCAACATCCTGACAGTCATCCAC 3′

Table S2. List of antibodies used in this study.

Antibody	Source	Catalog number	Dilution
β-ACTIN	Santa Cruz Biotechnologies	sc-1616	1:5000
HO-1	Chemicon International	AB1284	1:1000
LAMIN B	Santa Cruz Biotechnologies	sc-6217	1:1000
LC3	Sigma	L7543	1:1000
NRF2	Abyntek	AJ1555a	1:2000
NQO1	Abcam	ab2346	1:2000
p62	Abcam	ab56146	1:1000
TIMM23	BD Biosciences	611222	1:1000
